# German Post-War Election Database (GPWED)

**DOI:** 10.1038/s41597-025-06091-5

**Published:** 2025-10-27

**Authors:** Julian Voß

**Affiliations:** Osnabrück University, Osnabrück, Germany

**Keywords:** Politics, Government

## Abstract

Historical social science research on the electoral politics of transitional countries is often undermined by a lack of disaggregated, fine-grained election data. This paper presents the first national database of municipal-level results for one of the most notable cases of a transition to democracy in recent history: Post World War II West Germany. Spanning the country’s initial six federal elections between 1949 and 1969, the data covers significant historical developments such as the early phase of economic reconstruction and recovery, the social and political integration of millions of Germans expelled from the former eastern territories, the beginning judicial confrontation with the crimes of the Nazi regime, and the emergence of radical left-wing activism during the 1960s. The database will allow future research to address important questions on topics such as democratization, the dynamics of post-authoritarian and post-war politics, and the long-term evolution of electoral politics in one of Europe’s most populous nations.

## Background & Summary

Elections are central to democratic governance and have been shown to influence the trajectory of emerging democracies^1^. While recent research, especially within the growing field of Historical Political Economy (HPE), has intensified efforts to study the legacies of authoritarianism and democratization, such work often faces a core challenge: access to fine-grained historical data^2^. Since individual-level survey data is frequently unavailable for historical periods, studies of political attitudes and behavior in these contexts typically rely on aggregated information from small spatial units, which often represent the only viable basis for empirical analysis. Access to electoral data at granular spatial resolution is therefore essential to mitigate issues such as aggregation bias, while also enabling researchers to examine political dynamics that vary locally–such as community-specific historical experiences, local economic conditions, or subregional institutional differences.

Post-World War II Europe offers a particularly relevant context for this literature, with Germany standing out as a key example of a successful democratic transition^3^. Yet, detailed electoral data from this period has been difficult to obtain and prior research has been limited either to more aggregated units like counties^4^ or to regional case studies focusing on specific states^5^. The absence of centralized fine-grained election data for this period can be attributed to two circumstances. First, the publication of municipal-level election results in Germany is in the hands of state-level statistical offices. Although some German states have published these results in machine-readable formats, for other regions this information is only available in printed volumes. As the West German state was composed of more than 24000 independent municipalities during the 1950s and 1960s, the task of collecting, digitizing, and standardizing this large amount of data across different states has been a considerable obstacle. Second, many German states carried out territorial reforms in the 1960s and 1970s, leading to a substantial reduction in the number of independent municipalities. Therefore, many municipalities have been subject to one or even multiple mergers since the early postwar period, complicating the creation of election data at a consistent unit of analysis.

This paper introduces the first database of municipal-level results for federal parliamentary elections in West Germany during the early post-war years. In Germany, municipalities (Gemeinden) are the lowest level of government with elected councils and significant responsibilities in local governance. They are nested within counties (Landkreise) or function as independent cities (kreisfreie Städte). In contrast, federal electoral districts (Wahlkreise) are drawn solely for parliamentary and state elections and do not necessarily follow administrative boundaries. While counties manage broader regional services, municipalities are responsible for a wide spectrum of tasks–ranging from infrastructure and education to local economic development. This gives municipalities substantial political relevance and makes them a meaningful unit of analysis in electoral research.

I provide harmonized data to the boundaries of contemporary (2022) municipalities, enabling longitudinal panel analyses despite historical boundary changes due to municipal mergers and reforms. The dataset contains absolute vote counts for individual parties, the total number of valid votes, and the size of the eligible voting population per municipality. One caveat of the dataset is that, beginning in 1957, it does not include votes cast by mail-in voters, as these cannot be reliably assigned to individual municipalities for this period–affecting up to approx. 7% of ballots per election.

The dataset complements recent efforts like the GERDA project, which provides harmonized municipal-level electoral data for various types of elections since the early 1990s^6^. I extend this temporal coverage and provide information on electoral behavior for six federal elections between 1949 and 1969 covering important historical developments such as the country’s early phase of economic reconstruction and recovery, the social and political integration of millions of Germans expelled from the former eastern territories, the beginning judicial confrontation with the crimes of the Nazi regime, or the emergence of radical left-wing activism during the 1960s. It will allow future research to address important questions on topics such as democratization, post-authoritarian and post-war politics, and the long-term evolution of electoral politics in one of Europe’s most populous societies.

## Methods

### Collection

German federal elections represent a mixed-member proportional system and, since 1953, voters have two separate votes. With the first vote, voters decide between candidates in single-member districts, while with the second vote, voters choose between closed party lists at the state level. Since the distribution of seats in the German Bundestag depends directly on the share of party-list votes received by a party, this component of the ballot carries substantially more weight. Therefore, my data collection has focused on gathering information on party-list votes cast at the most granular level available, which in the case of German federal elections is the municipal level. Nevertheless, due to the fact that certain states release information regarding the results of both candidate and party-list votes together, I was able to collect data pertaining to candidate votes across several election years and states.

**Fig. 1:**
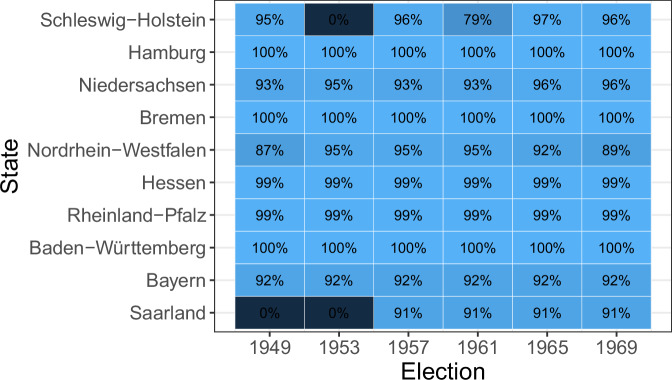
Database coverage. *Note:* The figure shows the availability of data on party-list votes by state and election year. Percentages indicate the proportion of non-missing municipality observations. Voters in West Berlin were not eligible to vote in federal elections until German reunification.

I contacted all relevant state statistical offices with requests for municipal-level election returns for federal parliamentary elections between 1949 and 1969. Five states (Baden-Württemberg, Bayern, Bremen, Hamburg, and Hessen) provided me with the complete series of results at the level of contemporary municipalities. For all other states (Niedersachsen, Nordrhein-Westfalen, Saarland, Schleswig-Holstein, and Rheinland-Pfalz), I either acquired PDF scans of the publications through the state statistical offices or the relevant publications were scanned by myself and two research assistants. Through this procedure, I could collect the relevant information for all remaining elections. Only the publication for the 1953 election in Schleswig-Holstein could neither be accessed through the state statistical office nor through any public library. Since the Saarland was under French occupation until 1956 and voters in this state have only been able to vote in federal elections since its accession in 1957, coverage for this state begins with the 1957 federal election. Figure 1 gives an overview of all election years covered by the database and the availability of information on party-list votes (See Appendix A for the coverage of candidate votes). The database includes a total of 82615 data points, representing 87% of all municipality-election-year observations between 1949 and 1969.

### Digitization

In those cases where the relevant election data was not available in a digital format, the statistical tables contained within the PDF scans had to be transformed into a structured, machine-readable format. For this purpose, I developed a digitization pipeline in Python and R. The complete code of this pipeline has been archived within a public GitHub repository (https://github.com/julian-voss/gpwed). I have processed 2950 pages of scans of statistical tables in total through this procedure, and the Appendix section D provides the full list of digitized sources. The pipeline involves the steps described below.

In a first step, each image scan is processed using a Python script based on the Amazon Textract API and the associated Textractor package. Although the Textract API offers table data extraction for document images, the results in my use case have been insufficient and contained high error rates. A central problem has been the incorrect recognition of table rows, preventing the correct assignment of data points to specific units. Therefore, only the predicted column layout information was used for further processing. Within each identified table column region, the Python script assigns the OCR text information to individual table cells based on a manually set parameter specifying the maximum allowed vertical distance between two text blocks of the same cell. Likewise, individual cells from different columns were then assigned to contiguous rows based on the maximum allowed vertical distance between two cells within the same row. Because the layout of municipality tables varies between state statistical offices and elections, different values for these parameters had to be set for different sets of tables manually.

For every statistical table scanned, the first step outputs a single Excel file. In a second step, all files are cleaned through a script written in R. Recurring cleaning steps include the (1) appropriate naming of columns and deletion of misidentified columns, (2) ensuring that data rows are correctly assigned to individual municipalities, (3) verifying the accuracy and correct assignment of information on higher-level administrative units, and (4) cleaning numeric columns for OCR errors like non-numerical characters or punctuation. The second step outputs a single data file for each state-election combination.

### Aggregation

In a third step, individual state files were merged into a single data file for every election, and the information is mapped to German municipalities as of 2022. In order to obtain unique identifiers for each historical municipality, the election tables are first merged with official municipality lists for the respective years provided by the federal statistical office^7^. Merging is performed through string matching based on county and municipality names using the LinkTransformer library and a pre-trained language model in order to account for inconsistencies due to different spellings of municipality names or OCR errors^8^. The correct matching of municipality pairs with low character similarity was then verified manually.

The majority of German municipalities have been subject to at least one merger since 1949. I rely on annual lists of name and territorial changes provided by the German Federal Statistical Office to create crosswalks for each election year linking historical municipalities to contemporary municipality boundaries^9^. I assign each historical municipality to a corresponding contemporary municipality, after which I aggregate all vote counts to the level of the modern municipality. Splits of municipalities have been relatively uncommon in the German context and typically result in one successor receiving the majority of the original municipality’s population. However, in those cases, I follow Roesel^10^ and assign the full vote count to the modern municipality that received the largest share of the historical population.

Before aggregating vote counts to contemporary municipalities, I address the issue that data can be missing for some municipalities due to errors in the OCR pipeline. For a specific election year, consider a historical municipality *i* that has been part of a historical county *j* and is now assigned to a modern municipality *k*. Let *p*_*ijk*_, *p*_*j*_ and *p*_*k*_ denote the historical population sizes of these entities.

Each modern municipality *k* is composed of a set of historical municipalities ${{\mathcal{H}}}_{k}=\{{h}_{1},\ldots ,{h}_{{n}_{k}}\}$. Let ${{\mathcal{H}}}_{k}^{obs}\subseteq {{\mathcal{H}}}_{k}$ be the set of historical municipalities with non-missing values. Similarly, let ${{\mathcal{C}}}_{j}=\{{c}_{1},\ldots ,{c}_{{m}_{j}}\}$ be the set of historical municipalities in county *j*, and ${{\mathcal{C}}}_{j}^{obs}\subseteq {{\mathcal{C}}}_{j}$ the subset with non-missing values. Then the observed population share of municipality *k* and county *j* is:

$${p}_{k}^{obs}=\frac{{\sum }_{h\in {{\mathcal{H}}}_{k}^{obs}}{p}_{h}}{{p}_{k}}\quad \quad {p}_{j}^{obs}=\frac{{\sum }_{c\in {{\mathcal{C}}}_{j}^{obs}}{p}_{c}}{{p}_{j}}$$

Let *y*_*ijk*_ denote the variable of interest for the historical municipality *i*. I employ the following imputation procedure in the cases of missing values:

$${\widehat{y}}_{ijk}=\{\begin{array}{ll}\frac{1}{| {{\mathcal{H}}}_{k}^{obs}| }{\sum }_{h\in {{\mathcal{H}}}_{k}^{obs}}{y}_{hjk} & if\,{p}_{k}^{obs} > 0.5\\ \frac{1}{| {{\mathcal{C}}}_{j}^{obs}| }{\sum }_{c\in {{\mathcal{C}}}_{j}^{obs}}{y}_{cjk} & if\,{p}_{k}^{obs}\le 0.5\,and\,{p}_{j}^{obs} > 0.5\\ missing & otherwise\end{array}$$

Only modern municipalities for which complete data is available after this imputation step are retained, and overall, only a small subset of municipalities is affected by the imputation method (See Table 1). To allow users to assess and possibly exclude imputed data from analyses, for each modern municipality, the final dataset includes information on the population-weighted share of historical municipalities whose values were imputed.

**Table 1:** Modern municipalities containing imputed voting information.

Election	Municipalities (%)
1949	6.38
1953	7.79
1957	7.49
1961	6.97
1965	3.56
1969	3.74

*Note:* The table presents information on the share of modern municipalities containing imputed voting information across election years.

#### Aggregated vote counts

For a limited number of states in certain election years, votes for smaller parties are not reported individually, but in an aggregated column combined with votes received by independent candidates. Table 2 provides a list of relevant years and parties.

**Table 2:** Aggregated vote shares.

State	Year	Parties included
Baden-Württemberg	1949	Kommunistische Partei Deutschlands (KPD)
Baden-Württemberg	1953	Deutsche Reichspartei (DRP)
Saarland	1961	Deutsche Friedens-Union (DFU), Gesamtdeutsche Partei Deutschlands (GDP), Deutsche Gemeinschaft (DG), Deutsche Reichspartei (DRP)
Niedersachsen	1965	Frei-Soziale Union (FSU), Aktionsgemeinschaft Unabhängiger Deutscher (AUD)
Saarland	1965	Deutsche Friedens-Union (DFU), Christlichdemokratische Volkspartei (CVP), Aktionsgemeinschaft Unabhängiger Deutscher (AUD)
Niedersachsen	1969	Europa Partei (EP), Frei-Soziale Union (FSU), Gesamtdeutsche Partei Deutschlands (GPD)

*Note:* The table lists cases in which the vote shares of smaller parties are given in aggregate.

#### Missing mail-in votes

Since 1957, voters in Germany have been able to vote by mail under certain conditions, and between 1957 and 1969, the share of mail-in voters ranged between 4.9% and 7.1%. With the exception of municipalities in Bavaria and independent cities in selected states, municipal-level election results generally do not include mail-in votes. The dataset therefore includes a variable indicating whether the reported results for a municipality incorporate mail-in voting. Since mail voters tend to differ systematically from in-person voters, analyses based solely on in-person votes may be subject to measurement error. To assess the magnitude of this potential bias, I draw on county-level data from the state of Hesse (see Appendix C.2). The analysis suggests that the extent of measurement error introduced by the exclusion of mail-in voters is generally small and typically within  ±0.5 percentage points for most counties examined. Nevertheless, users should be aware of this issue. The share of mail-in voters varies both over time and across municipalities and is likely correlated with other relevant factors, potentially affecting inferential analyses. Moreover, this limitation implies that turnover rates can only be reliably calculated for the first two elections.

#### Missing values

**Fig. 2:**
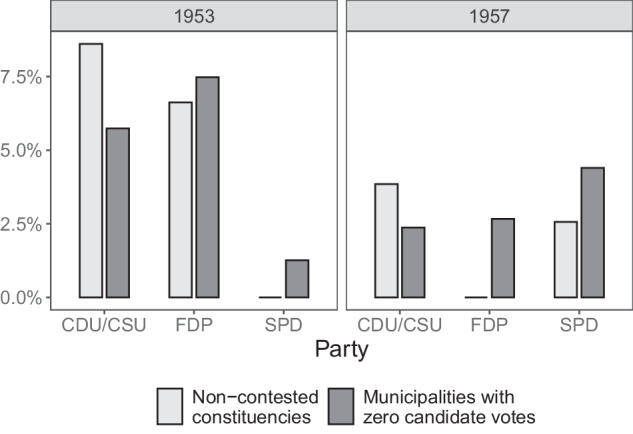
Districts without party candidates and zero-vote municipalities. *Note:* This figure shows the share of constituencies in which the major German parties did not field a direct candidate (light grey bars), as well as the share of municipality-level observations in the GPWED with zero recorded candidate votes. Statistics on non-contested constituencies are based on data by the Federal Returning Officer^17^. The data are shown for the 1953 and 1957 federal elections only as since 1961, the respective parties have nominated candidates in all districts.

Users should be aware of how missing values are coded in the data. In valid rows, a missing value for party-list votes indicates that the party did not run in the relevant state and election year. When considering candidate votes, a missing value indicates that no candidate of the particular party received any votes in the relevant state and election year. It is not possible to distinguish whether a value of zero for party-list votes indicates that no candidate of the specific party has received votes in a given municipality or that the party has not put up a candidate in the relevant constituency. For the three major German political parties, this issue is only relevant for the 1953 and 1957 elections, as between 1961 and 1969 the CDU/CSU, SPD, and FDP fielded direct candidates in all constituencies. Figure 2 illustrates the share of uncontested constituencies and municipalities reporting zero votes for the respective parties, highlighting that this issue affects only a small subset of municipalities. However, researchers may pay particular attention to this when analyzing smaller parties, which often fielded direct candidates only in specific regions.

## Data Records

The data has been published through an Open Science Framework (OSF) repository^11^. The repository contains a Readme file and the complete database in three different commonly used data formats. Additionally, users can access the dataset by installing the accompanying R package directly from GitHub (https://github.com/julian-voss/gpwed). Table 3 lists the variables available in the data set. The eight-digit municipality key uniquely identifies every German municipality and allows users to link the information to other sources. In addition to information on electoral returns for individual parties and the total number of votes, the data also includes information on the size of the eligible voting population per municipality.

**Table 3:** Variables.

Variable	Description
key_state	Two-digit state identifier
ags	Eight-digit municipality identifier
municipality_name	Name of municipality
independent_city	Binary indicator for independent cities (Kreisfreie Städte)
year	Election year
vote_type	Type of vote
share_imputed	Share of imputed values
voters_eligible	No. of eligible voters
votes_valid	No. of valid votes
*Party vote counts*	Different variables capturing party vote counts
cdu_county_deviation	Binary indicators for county-level deviations > 1%
spd_county_deviation	
fdp_county_deviation	
mail_votes	Binary indicator for the inclusion of mail-in votes

*Note:* This table lists the variables available in the GPWED data set. See Table B.1 in the Appendix for a complete list of party variables.

**Fig. 3:**
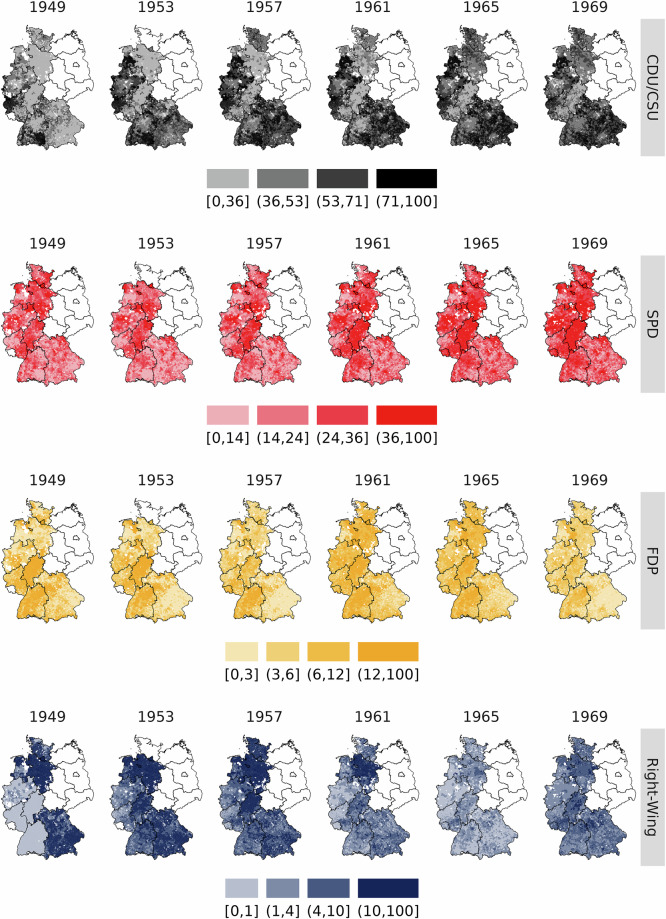
Municipal-level vote shares of major parties, 1949–1969. *Note:* This Figure visualizes municipal-level (party) vote shares of the three main German political parties as well as all right-wing nationalist parties for all elections covered by the data set. Table 4 lists all individual parties grouped as right-wing nationalist parties. Color scales represent party-specific quartiles.

Figure 3 offers a snapshot of the data set by mapping trends in municipal-level (party) vote shares over time for Germany’s main political parties: the conservative CDU/CSU alliance, the social-democratic SPD, and the liberal FDP. In addition, the figure also shows the combined vote share for right-wing nationalist parties. Table 4 lists all parties classified as having a right-wing nationalist platform.

**Table 4:** List of right-wing nationalist parties.

Party	Active Years
Wirtschaftliche Aufbau-Vereinigung (WAV)	1949
Deutsche Partei (DP)	1949, 1953, 1957
Deutsche Reichspartei (DRP)	1949, 1953, 1957, 1961
Gesamtdeutsche Block / Bund der Heimatvertriebenen und Entrechteten (GB/BHE)	1953, 1957
Partei der guten Deutschen / Dachverband der Nationalen Sammlung (PDGD/DNS)	1953
Vaterländische Union (VU)	1953, 1957
Deutsche Gemeinschaft (DG)	1957, 1961
Gesamtdeutsche Partei (GPD)	1961, 1969
Nationaldemokratische Partei Deutschlands (NPD)	1965, 1969
Unabhängige Arbeiter-Partei (UAP)	1965, 1969

*Note:* This table list all parties defined as right-wing nationalist for all supplementary analyses. The classification of parties is based on Stöss^16^.

## Technical Validation

To ensure the validity of the curated data, I perform a number of validity checks. First, during the digitization process, I assess whether the information on vote counts meets two numerical constraints. I discard all observations for historical municipalities in which the sum of reported votes for individual parties deviates from the indicated number of valid votes by more than  ±1% or where the number of valid votes exceeds the size of the eligible voting population.

Second, I compare the curated data with official county-level election returns. For federal elections between 1953 and 1969, the German Federal Statistical Office has published county-level results, which at least include vote counts for the three main political parties, as well as an aggregated category for all other parties. To assess to what degree the generated data matches official returns, I calculate differences in county-level vote shares for the CDU/CSU, SPD, FDP, and all other parties. Information on the distribution of deviations is presented in Table 5. The table shows that discrepancies between the curated data and official returns are only marginal. Overall, deviations at the county level remain below  ±1% for 93% of all observations.

**Table 5:** County-level deviations by Party.

Party	Mean	SD	Min	*P*_25_	Median	*P*_75_	Max
CDU/CSU	0.11	0.86	−3.70	−0.11	0	0.32	11.02
SPD	−0.10	0.83	−8.67	−0.24	0	0.13	3.60
FDP	−0.03	0.34	−2.49	−0.09	0	0.05	2.72
Other	0.00	0.33	−1.98	−0.05	0	0.03	3.32

*Note:* This table shows summary statistics of differences in county-level vote shares (p.p.) between the curated data and official administrative returns provided by the Federal Returning Officer. I compare vote shares for the three main political parties (CDU, SPD, and FDP) and all other parties.

**Fig. 4:**
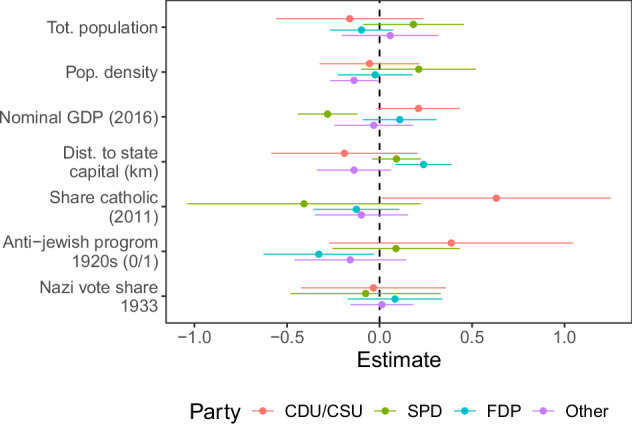
County-level deviations and socio-economic characteristics. *Note:* This figure shows estimates from OLS regressions of county-level deviations in party vote shares on several socio-economic covariates. Covariates are taken from^13,14^. All continuous variables are standardized and models include state and year fixed effects. Error bars represent 95% confidence intervals based on standard errors clustered at the state level.

In order to determine the extent to which remaining measurement errors are systematic in nature, I compare deviations at the county level to a number of socio-economic factors. I use data from Erfort^12^ who has mapped the historical election data provided by the Federal Statistical Office to present-day counties using area-weighted interpolation and combine this with several contemporary and historical covariates taken from^13,14^. I estimate OLS regressions of county-level deviations in party vote shares on the set of available covariates. Figure 4 presents the results of this exercise. Overall, the estimated coefficients are mostly insignificant and small in size, indicating that the proportion of systematic measurement error in the data seems to be close to zero. While a few coefficients turn out significant, their substantive size remains marginal. For example, the reported 0.63 percentage point overestimation of the Conservative Party’s electoral support in areas with higher Catholic share amounts to only about 0.04 standard deviations of that party’s vote share. Furthermore, I cannot rule out whether these errors stem from the interpolation method used to generate the benchmark data. As is the case with most social science data, users should be aware of this potential for measurement error, e.g. especially when evaluating small effect sizes^15^.

## Usage Notes

### Differences to GERDA

Users may be particularly interested in combining the data with the recent GERDA database^6^. While GERDA uses harmonized municipal boundaries as of 2021, the GPWED is harmonized to the 2022 administrative boundaries. The differences between the 2021 and 2022 boundary schemes are minimal: only two municipalities in western Germany have been affected by mergers or boundary changes between those two years.

Both datasets follow a conceptually similar crosswalking approach: historical election results are linked to contemporary municipalities by mapping predecessor units to their successor entities. However, the method used here adopts a simplified procedure: it ignores rare cases of municipal splits and assigns all votes to the contemporary municipality that absorbed the largest share of the historical unit (see above for details). Lastly, due to data limitations, the GPWED does not include votes cast by mail-in voters.

In sum, both datasets are largely compatible and can be merged for long-run analyses at the municipal level. However, depending on the research design, users should be mindful of differences in crosswalking procedures and the exclusion of mail-in votes in GPWED, as these may have implications for certain types of analyses.

## Supplementary information

Supplementary information

## Data Availability

The complete code used to create the database has been archived in a public GitHub repository: https://github.com/julian-voss/gpwed.

## Data availability

The data has been published through an Open Science Framework (OSF) repository^11^. The repository contains a Readme file and the complete database in three different commonly used data formats. Additionally, users can access the dataset by installing the accompanying R package directly from GitHub (https://github.com/julian-voss/gpwed).
