# Supplementary material for: German Post-War Election Database (GPWED)
Source: Sci Data. 2025 Oct 27;12:1698. doi: 10.1038/s41597-025-06091-5 (PMC12559339; doi:10.1038/s41597-025-06091-5)
Supplement: Supplementary file 1 — Supplementary information [file 41597_2025_6091_MOESM1_ESM.pdf]

German Post-War Election Database

Online Appendix

Table of Contents

A Candidate votes **1**

B Party variables **2**

C Validation **4**

C.1 County-level deviations before imputation 4

C.2 Missing mail-in votes 5

D Sources **7**

A Candidate votes

Figure A.1: Database coverage

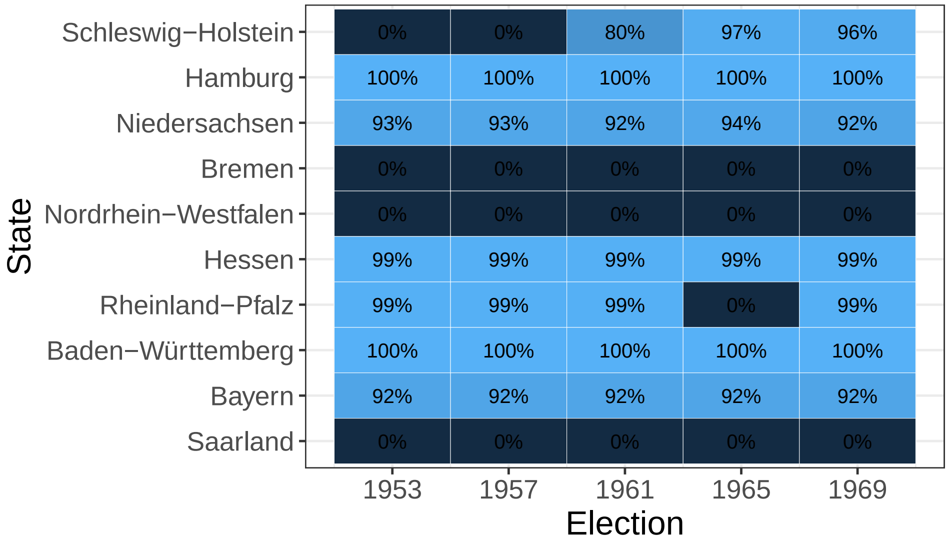

Note: The figure shows the availability of data on candidate votes by state and election year. Percentages indicate the proportion of non-missing municipality observations. Voters in West Berlin were not eligible to vote in federal elections until German reunification. The state of Saarland was under French occupation until 1956 and voters in this state have only been able to vote in federal elections since its accession in 1957.

B Party variables

Table B.1: List of party variables

Variable	Parties included	Relevant years / states
cdu_csu	Christlich Demokratische Union Deutschlands, Christlich-Soziale Union in Bayern	All
spd	Sozialdemokratische Partei Deutschlands	All
fdp	Freie Demokratische Partei	All
ap	Arbeiterpartei	1949 (HE)
bp	Bayernpartei	1949 (BY), 1953 (BY), 1969 (BY)
center_fu	Deutsche Zentrumspartei, Föderalistische Union	1949 (SH, NI, NRW), 1953 (NRW), 1957 (NI, NRW, BY), 1969 (NRW, SA)
dp	Deutsche Partei	1949 (NI, SH, NRW), 1953, 1957
drp	Deutsche Konservative Partei – Deutsche Rechtspartei, Deutsche Reichspartei	1949 (NI, SH, NRW, HH), 1953 (Except HE), 1957, 1961
kpd	Kommunistische Partei Deutschlands	1949, 1953
rsf	Radikal-Soziale Freiheitspartei	1949 (NI, SH, NRW, HH, HB)
rvwp	Rheinisch-Westfälische Volkspartei	1949 (NRW)
ssw	Südschleswigscher Wählerverband	1949 (SH), 1957 (SH), 1961 (SH)
wav	Wirtschaftliche Aufbau-Vereinigung	1949 (BY)
gb_bhe	Gesamtdeutsche Block / Bund der Heimatvertriebenen und Entrechteten	1953, 1957
gvp	Gesamtdeutsche Volkspartei	1953
pdgd_dns	Partei der guten Deutschen, Dachverband der Nationalen Sammlung	1953 (HE, BY, BW)
vu	Vaterländische Union	1953 (BY), 1957
bdd	Bund der Deutschen, Partei für Einheit, Frieden und Freiheit	1957
dg	Deutsche Gemeinschaft	1957 (Except SH, NRW, HB), 1961 (Except HH, HB)
dms	Deutscher Mittelstand	1957 (NI, NRW, BY)
cvp	Christlichdemokratische Volkspartei	1957 (SA), 1965 (NRW, SA)
dfu	Deutsche Friedens-Union	1961, 1965
gpd	Gesamtdeutsche Partei, Gesamtdeutsche Partei Deutschlands	1961, 1969 (SH, NRW, HE, BY, BW)
npd	Nationaldemokratische Partei Deutschlands	1965, 1969
aud	Aktionsgemeinschaft Unabhängiger Deutscher	1965

Continued on next page

Table B.1: List of party variables (continued)

Variable	Parties included	Relevant years / states
fsu	Frei-Soziale Union	1965 (Except RP, HE, HB, SA), 1969 (Except RP, HE, SA)
uap	Unabhängige Arbeiter-Partei	1965 (NRW), 1969 (NRW, HE)
adf	Aktion Demokratischer Fortschritt	1969
ep	Europa Partei / Europäische Föderalistische Partei	1969 (Except RP)
other	Other parties / independent candidates	All

Note: This table lists all party variables present in the data set and the respective parties covered by individual variables. Unless otherwise stated, the listing of an election year means that the party ran with its own list in all federal states.

C Validation

C.1 County-level deviations before imputation

Table C.2 presents information on deviations in county-level vote shares between the curated data and official administrative returns, excluding municipalities for which voting data has been imputed.

Table C.2: County-level deviations by Party

Party	Mean	SD	Min	P_{25}	Median	P_{75}	Max
CDU/CSU	0.13	1.10	-5.79	-0.14	0	0.35	10.99
SPD	-0.12	1.01	-10.69	-0.28	0	0.16	4.67
FDP	-0.03	0.38	-2.50	-0.10	0	0.07	3.21
Other	0.00	0.43	-3.47	-0.06	0	0.04	5.52

Note: This table shows summary statistics of differences in county-level vote shares (p.p.) between the curated data and official administrative returns provided by the federal statistical office. I compare vote shares for the three main political parties (CDU, SPD, and FDP) and all other parties.

C.2 Missing mail-in votes

To quantify the degree of measurement error introduced by discarding mail-in voters, I use county-level election returns for the state of Hesse between 1957 and 1969. Specifically, for each county c, party p, and election year t, I compute the error as the difference between a party's vote share based on in-person (urn) votes and the overall vote share (including mail-in votes):

$$\varepsilon_{cpt} = V_{cpt}^{\text{urn}} - V_{cpt}^{\text{total}} \quad (1)$$

where V_{cpt}^{urn} is a party's vote share among in-voters and V_{cpt}^{total} is the vote share among all voters (including mail-in voters).

Figure C.2 visualizes the distribution of these deviations by election year. The figure shows that the level of error introduced by omitting mail-in voters is generally small and below ±1 percentage points. Across election years, more than 90% of the county-party observations exhibit deviations within ±0.5 percentage points.

Figure C.2: Distribution of errors from omitting mail-in votes

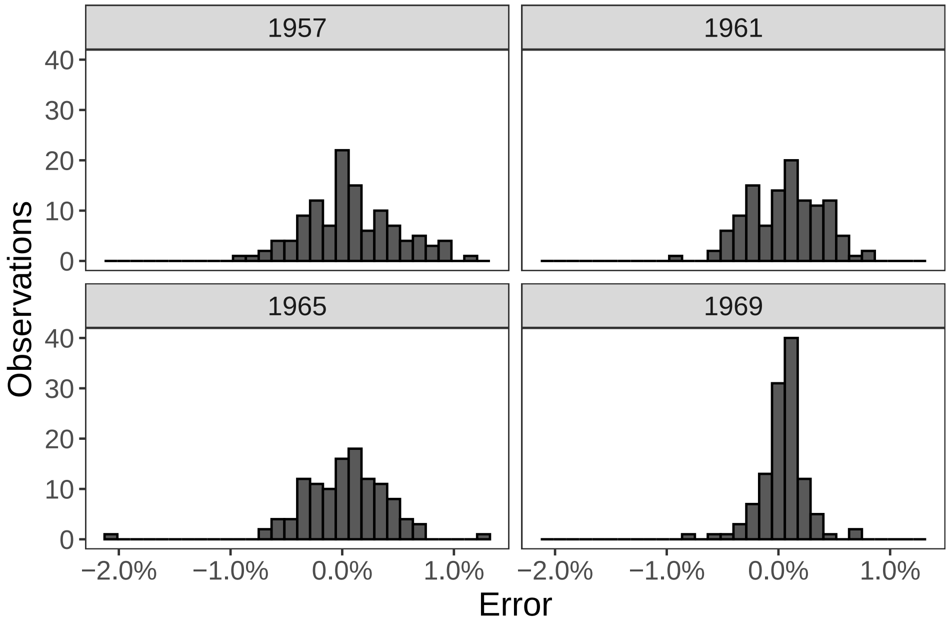

Note: This figure visualizes the distribution of potential measurement error ε_{cpt} introduced by discarding mail-in votes for the state of Hesse. Positive values indicate an overestimation of a party's county-level vote share.

Table C.3 summarizes the distribution of deviations by party. For the state of Hesse, discarding mail-in votes leads to a slight overestimation for the CDU and FDP while the SPD's vote share is slightly underestimated.

Table C.3: Errors from omitting mail-in votes by party

Party	Mean	SD	Min	P_{25}	Median	P_{75}	Max
CDU	0.37	0.25	-0.71	0.21	0.34	0.48	1.26
SPD	-0.28	0.25	-2.07	-0.38	-0.26	-0.15	0.56
FDP	0.07	0.09	-0.19	0.03	0.08	0.13	0.49
Other	-0.01	0.04	-0.22	-0.02	0.00	0.01	0.24

Note: The table summarizes the distribution of deviations ε_{cpt} by party.

D Sources

1. Nordrhein-Westfalen, S. L. *Die Wahlen in Nordrhein-Westfalen seit 1948* (Düsseldorf, 1952)

2. Nordrhein-Westfalen, S. L. *Die Bundestagswahl in Nordrhein-Westfalen. 6. September 1953* (Düsseldorf, 1954)

3. Nordrhein-Westfalen, S. L. *Die Bundestagswahl am 15. September 1957 in Nordrhein-Westfalen* 85 (Düsseldorf, 1958)

4. Nordrhein-Westfalen, S. L. *Die Bundestagswahl am 17. September 1961 in Nordrhein-Westfalen* 150 (Düsseldorf, 1962)

5. Nordrhein-Westfalen, S. L. *Die Wahl zum 5. Deutschen Bundestag am 19. September 1965 in Nordrhein-Westfalen* B III 1-65 (Düsseldorf, 1966)

6. Nordrhein-Westfalen, S. L. *Bundestagswahl 1969. Ergebnisse nach Wahlkreisen und Gemeinden.* (Düsseldorf, 1970)

7. Rheinland-Pfalz, S. L. *Die Wahl zum ersten Bundestag am 14. August 1949* 4 (Bad Ems)

8. Rheinland-Pfalz, S. L. *Die Wahl zum zweiten Bundestag am 6. September 1953* 29 (Bad Ems, 1953)

9. Rheinland-Pfalz, S. L. *Die Wahl zum zweiten Bundestag am 6. September 1957* 53 (Bad Ems, 1958)

10. Rheinland-Pfalz, S. L. *Die Wahl zum vierten Bundestag am 17. September 1961* 94 (Bad Ems, 1961)

11. Rheinland-Pfalz, S. L. *Die Wahl zum vierten Bundestag am 17. September 1969* 203 (Bad Ems, 1969)

12. des Saarlandes, S. A. *Die Wahlen im Saarland am 4. Dezember 1960* 17 (Saarbrücken, 1961)

13. des Saarlandes, S. A. *Die endgültigen Ergebnisse der Bundestagswahl am 17. September 1961* B III 1-j-1/61 (Saarbrücken, 1961)

14. des Saarlandes, S. A. *Endgültige Ergebnisse der Bundestagswahl im Saarland am 28. September 1969* 60 (Saarbrücken, 1969)

15. Schleswig-Holstein, S. L. *Die Wahl zum ersten Bundestag in Schleswig-Holstein* D (Kiel, 1950)

16. Schleswig-Holstein, S. L. *Die Wahl zum 3. Deutschen Bundestag am 15.9.1957 in Schleswig-Holstein (Endgültiges Ergebnis)* B III 1-3/1957 (Kiel, 1957)

17. Schleswig-Holstein, S. L. *Die Wahl zum 4. Deutschen Bundestag am 17.9.1961 in Schleswig-Holstein (Endgültiges Ergebnis)* B III 1-5/1961 (Kiel, 1961)

18. Schleswig-Holstein, S. L. *Die Bundestagswahl am 19. September 1965 in Schleswig-Holstein. Endgültiges Ergebnis.* B III 1-5/1965 (Kiel, 1965)

19. Schleswig-Holstein, S. L. *Die Bundestagswahl am 19. September 1969 in Schleswig-Holstein. Endgültiges Ergebnis.* B III 1-5/69 (Kiel, 1969)

20. Statistik, N. L. .-. *Die Ergebnisse der Wahl zum 1. Bundestag am 14. August 1949 nach Verwaltungsbezirken* (Hannover)

21. Statistik, N. L. .-. *Die Ergebnisse der Bundestagswahl 1949 in den Kreisangehörigen Gemeinden* (Hannover)

22. für Landesplanung und Statistik, N. A. *Die Wahl zum 2. Deutschen Bundestag in Niedersachsen am 6. September 1953* (Hannover, 1954)

23. für Landesplanung und Statistik, N. A. *Die Wahl zum 3. Deutschen Bundestag in Niedersachsen am 15. September 1957* (Hannover, 1958)

24. Statistik, N. L. .-. *Die Wahl zum 4. Deutschen Bundestag am 17. September 1961 in Niedersachsen.* (Hannover, 1962)

25. Statistik, N. L. .-. *Die Wahl zum 5. Deutschen Bundestag am 19. September 1965 in Niedersachsen* 80 (Hannover, 1966)

26. Statistik, N. L. .-. *Die Wahl zum 6. Deutschen Bundestag am 28. September 1969 in Niedersachsen. Teil 2. Wahlergebnisse in den Gemeinden und Ergebnisse der Repräsentativstatistik.* 133 (Hannover, 1970)
